# A Pilot Study for the Feasibility of Exome-Sequencing in Circulating Tumor Cells Versus Single Metastatic Biopsies in Breast Cancer

**DOI:** 10.3390/ijms21144826

**Published:** 2020-07-08

**Authors:** Pushpinder Kaur, Daniel Campo, Tania B. Porras, Alexander Ring, Janice Lu, Yvonne Chairez, Yunyun Su, Irene Kang, Julie E. Lang

**Affiliations:** 1 Department of Surgery, Keck School of Medicine, University of Southern California, Los Angeles, CA 90033, USA; Pushpinder.Bains@med.usc.edu (P.K.); yunyun.su@med.usc.edu (Y.S.); 2 University of Southern California Norris Comprehensive Cancer Center, Los Angeles, CA 90033, USA; janice.lu@med.usc.edu (J.L.); irene.kang@med.usc.edu (I.K.); 3 Department of Biological Sciences, University of Southern California, Los Angeles, CA 90089, USA; dcampof@gmail.com; 4 Cancer and Blood Disease Institute, Children Hospital Los Angeles, University of Southern California, Los Angeles, CA 90027, USA; porrastb@gmail.com; 5 Department of Oncology and Hematology, UniversitätsSpital Zürich, Rämistrasse 100, 8091 Zürich, Switzerland; alexander.ring@usz.ch; 6 Division of Medical Oncology, Department of Medicine and University of Southern California Norris Cancer Center, University of Southern California, Los Angeles, CA 90033, USA; 7 Department of Stem Cell Biology and Regenerative Medicine, Keck School of Medicine, University of Southern California, Los Angeles, CA 90033, USA; yvonne.chairez@med.usc.edu

**Keywords:** breast cancer, circulating tumor cells, metastasis, whole exome sequencing, clinically actionable, whole genome amplification

## Abstract

The comparison of the landscape of somatic alterations in circulating tumor cells (CTCs) versus metastases is challenging. Here, we comprehensively characterized the somatic landscape in bulk (amplified and non-amplified), spike-in breast cancer cells, CTCs, and metastases from breast cancer patients using whole-exome sequencing (WES). We determined the level of genomic concordance for somatic nucleotide variants (SNVs), copy number alterations (CNAs), and structural variants (SVs). The variant allele fractions (VAFs) of somatic variants were remarkably similar between amplified and non-amplified cell line samples as technical replicates. In clinical samples, a significant fraction of somatic variants had low VAFs in CTCs compared to metastases. The most frequently recurrent gene mutations in clinical samples were associated with an elevated C > T mutational signature. We found complex rearrangement patterns including intra- and inter-chromosomal rearrangements, singleton, and recurrent gene fusions, and tandem duplications. We observed high molecular discordance for somatic alterations between paired samples consistent with marked heterogeneity of the somatic landscape. The most prevalent copy number calls were focal deletion events in CTCs and metastases. Our results demonstrate the feasibility of an integrated workflow for the identification of a complete repertoire of somatic alterations and highlight the intrapatient genomic differences that occur between CTCs and metastases.

## 1. Introduction

The emerging field of cancer precision medicine infers that comprehensive molecular profiling of a patient’s tumor is required to detect actionable alterations for the selection of targeted therapy. Recent advances in next-generation sequencing (NGS)-based diagnostic technologies and bioinformatics capabilities have enabled physicians to provide patients with more personalized care. Although precision medicine has had some successes, the open-label randomized controlled SHIVA trial found that the use of molecularly targeted agents outside of their indications did not improve survival [1,2]. This can be due to intra-tumoral heterogeneity, spaciotemporal heterogeneity, or the fact that a tissue biopsy from the primary or single-site metastatic tumor biopsy are unlikely to contain the entire spectrum of mutations. This could also lead to sampling bias which may influence therapeutic decision-making [3]. To date, comprehensive molecular profiling has been performed on fresh/frozen and formalin-fixed paraffin-embedded (FFPE) samples from both primary and metastatic tumors [4]. However, the quality, quantity, and frequent unavailability of tumor specimens has become a significant barrier in the implementation of precision medicine into clinical practice. The use of liquid biopsies (e.g., circulating tumor cells (CTCs) or circulating tumor DNA (ctDNA)) represents an appealing alternative sample type due to being obtainable from a minimally invasive blood draw and amenable to serial repetition over time while also addressing some of the technical challenges [5,6,7]. The molecular analysis of CTCs also provides an effective means of monitoring disease progression during tumor evolution and to detect genomic aberrations present in tumor subclones at distinct metastatic lesions [8]. Moreover, CTCs have a higher level of heterogeneity that might assist in unveiling oncogenic drivers and explaining therapeutic drug resistance [9]. 

Many studies have shown that CTCs enumeration before and after treatment independently predicts for progression-free survival (PFS) and overall survival (OS) in metastatic breast cancer (MBC) patients [10,11]. Beyond enumeration, recent advances in NGS technologies have enabled the comprehensive analysis of bulk and single CTCs that provides critical insights into profiling breast cancer metastasis [2,12]. However, such analysis is challenging due to their extreme scarcity and low purity with most CTC enrichment methods. Since CTCs are extremely rare (a few CTCs/mL) in comparison to normal blood cells [13,14,15,16,17], extensive enrichment is necessary to construct sequencing libraries that may introduce amplification bias by polymerase errors. Single cell-studies have helped, to some extent, to identify methods that result in high genomic coverage and to distinguish real single nucleotide variants (SNVs) from artifacts [18,19]. The accurate detection of somatic variants in CTCs is very challenging as white blood cells (WBCs) or other background cell populations confound variant identification. Indeed, recent studies in MBC and other cancers have detailed the mutational landscape of CTCs isolated from the platforms that do not fix CTCs. However, most of the CTC DNA profiling methods used low-coverage (or low-pass) whole genome sequencing (WGS), targeted, or Sanger sequencing to identify genomic alterations in a panel of genes of interest [20,21,22,23]. 

The objective of our study was to evaluate the feasibility of combining whole genome amplification (WGA) with hybridization capture-based whole exome sequencing (WES) workflow, with maximal coverage for the regions of ~4600 medically relevant genes, in a breast cancer cell line spike-in experiment. This gene set includes the genes present in ClinVar and GeneTests consensus coding sequencing (CCDS) databases and assembled by the consortium of Emory Genetics lab, Harvard Laboratory of Molecular Medicine, and Children’s Hospital of Philadelphia (CHOP). After establishing feasibility in technical replicates using cell lines, we validated this workflow on MBC samples by sequencing both low-quantity (CTCs) and low-quality (FFPE) samples for the identification of clinically relevant alterations and to also determine the concordance of somatic alterations between CTCs and matched metastatic FFPE samples.

## 2. Results

### 2.1. Optimization of WES in Spiked and Unspiked Breast Cancer Cells

We first evaluated the technical feasibility of combining WGA with hybridization capture-based WES in identifying genomic variants in experimental samples. To mimic clinical CTC samples and to test the compatibility of the exome capture enrichment method with a low number of cells, the whole workflow was first optimized on spike-in samples as CTC mimics. Triple negative MDA-MB-231 breast cancer cells (*n* = 50) were spiked into a healthy donor blood sample in a Streck tube. WGA was performed with a multiple displacement amplification (MDA) based Repli-g single cell amplification kit in spike-in samples (Parsortix harvested), WBCs (healthy donor), and bulk cancer cells (MDA-MB-231 cells). Since WGA results in artifactual variants, we also included non-amplified gDNA (no Repli-g WGA) from MDA-MB-231 bulk cancer cells to determine the concordance of the variant detection in amplified vs. non-amplified samples. For bulk cancer cells, 10,000 MDA-MB-231 cells were used for DNA isolation. Given that a single cell contains approximately ~7pg of DNA, we estimated that we used 5 cells from bulk cancer cells and >1 cell from spike-in samples for sequencing. To assess the variant detection performance of WES Medexome assay and the reproducibility of the method, we performed the experiment in duplicates (WGA = spike-in samples (S1 and S2), WBCs (WL1 and WL2), and MDA-MB-231 bulk cancer cells (P1 and P2); non-WGA sample = MDA-MB-231 bulk cancer cells (MDA1 and MDA2)). 

**Figure 1:**
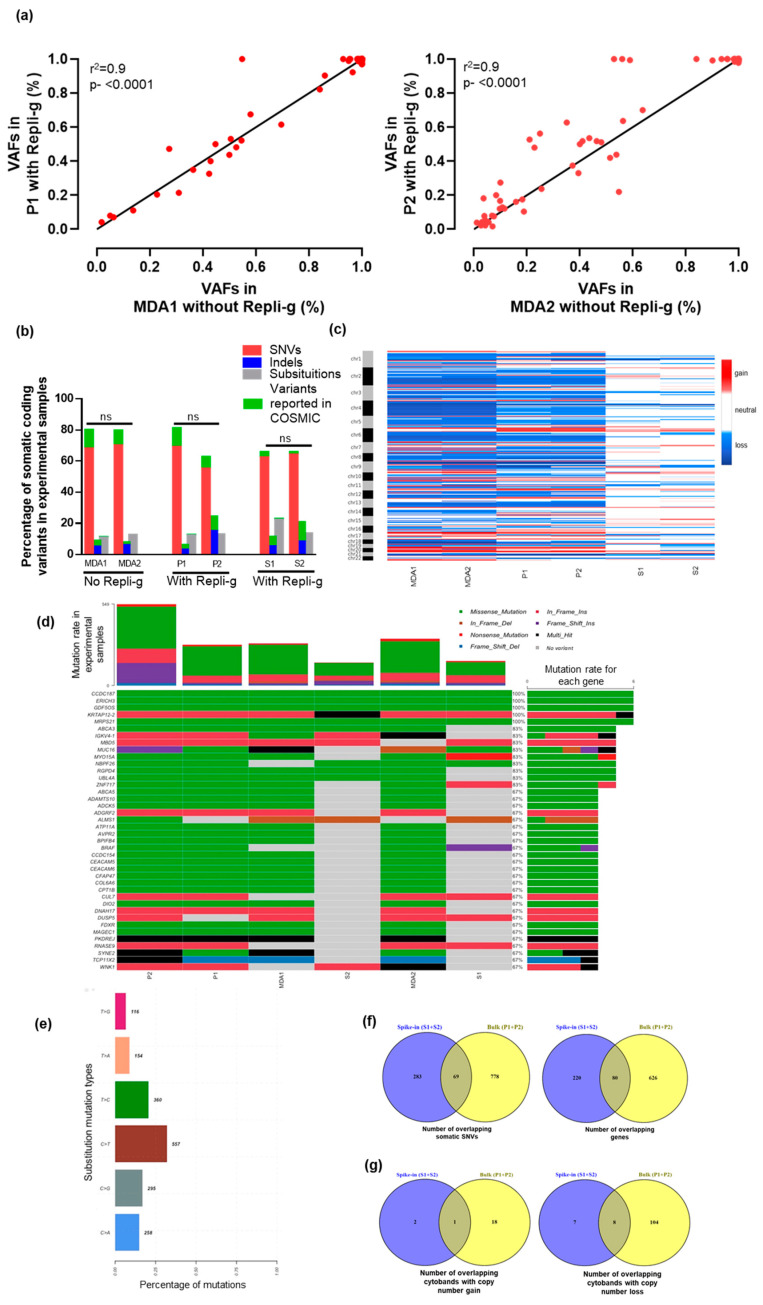
Overview of whole exome sequencing (WES) analysis in spike-in and unspiked breast cancer cells (**a**) Scatter plot of variant allele fractions (VAFs) detected using WES for replicate 1 and 2 of amplified and non-amplified bulk cancer MDA-MB-231 cells. For replicate 1, data points are for 133 shared variants between P1 and MDA1 (Pearson’s r^2^ = 0.9) and 163 shared variants for P2 and MDA2 (Pearson’s r^2^ = 0.9). (**b**) Bar graph depicting the percentage of coding variants in experimental samples (Bulk cells = MDA1 and MDA2 without Repli-g; Bulk cells = P1 and P2 with Repli-g; and, spike-in cells= S1 and S2 with Repli-g). The Mann–Whitney test was used to evaluate the differences between coding variants within technical replicates. (**c**) Copy number alteration (CNA) profile heatmap for amplified (P1, P2, S1, and S2) and non-amplified samples (MDA1 and MDA2) with gain (red), loss (blue), and neutral (white). Both Repli-g amplified DNA and non-amplified DNA showed a similar pattern of copy number profiles. The Pearson r test was used for correlation analysis between amplified and non-amplified samples. (**d**) Oncoplot showing the 40 most frequently mutated genes color-coded by type of mutations in experimental samples. The percentage to the right of the oncoplot shows the percent of samples with variants for the corresponding gene. Right, barplot shows the mutation rate in each of 40 mutated genes; Top, barplot shows the mutation rate for each patient for 40 most frequently mutated genes. By default, samples are ordered by the most frequently mutated genes. (**e**) Distribution of base substitutions in experimental samples revealed a signature characterized predominantly by C > T transition substitutions. The percent mutations are shown on the x-axis and substitution mutation types are on the y-axis. (**f**) The number of overlapping somatic single nucleotide variants (SNVs) and genes in Repli-g amplified bulk MDA-MB-231 (P1 + P2) and spike-in samples (S1 + S2). (**g**) The number of overlapping chromosomes cytobands (cytogenic bands) with copy number gains and losses in Repli-g amplified bulk MDA-MB-231 (P1 + P2) and spike-in samples (S1 + S2).

To assess the performance of the capture process and enrichment efficiency, we examined the percentage of target bases covered at 1x and 20x coverage thresholds. The amplified and non-amplified samples showed similar concordance for the on-target reads in both replicates, indicating high enrichment efficiency in experimental samples. Table 1 shows a summary of sequencing and alignment statistics for experimental samples. We observed no apparent differences for the percentage of on-target reads between low quantity samples and bulk cancer cells (P1 (80.7%) vs. S1 (77.6%) and P2 (79.6%) vs. S2 (78.5%)). The average overall sequence quality score was above 30 indicating a substantial number of high-quality bases in experimental samples. We next compared the variant allele fractions (VAFs) within the two technical replicates (P1 vs. MDA1 and P2 vs. MDA2). A significant correlation was observed for 133 shared variants in P1 vs. MDA1 (Pearson’s r^2^ = 0.98, *p* < 0.0001, two-tailed) and 163 variants in P2 vs. MDA2 (Pearson’s r^2^ = 0.95, *p* < 0.0001, two-tailed) (Figure 1a). The technical replicates of MDA-MB-231 cells showed r^2^ of 0.9, comparing with Repli-g versus without Repli-g, suggesting that the Repli-g WGA does not distort the relative proportion of various mutation types detected. Importantly, amplified MDA-MB-231 bulk cancer cells also revealed the presence of 4/5 variants reported by the American Type Culture Collection (ATCC) (BRAF (p.G464V), KRAS (p.G13D), NF2 (p.E231*), and TP53 (p.R280K)) [24]. Additionally, the variant overlap between MDA-MB-231 amplified and non-amplified bulk cancer cells included many oncogenes and tumor suppressors such as FAM83B, KRAS, APC, TP53, NF1, NF2, and MLH1 as well as other genes present in the Cancer Gene Census such as BARD1 and FBLN2 [25,26,27]. The variant allele fractions were also 100% for heterozygous mutations in genes such as TP53 (p.R241K; p.R148K; p.R269K; p.R280K; p.R121K), NF1 (p.T467fs*3), AR (p.T661T; p.T129T; p.T471T), and BRAF (p.G504V; p.G464V). We also found nearly similar concordance in the frequencies of protein-coding variants in amplified and non-amplified bulk cancer cells, with some of them being reported in the Catalogue of Somatic Mutations in Cancer (COSMIC) (Figure 1b). MDA-MB-231 cells are known to harbor more copy number losses than gains [28,29]. We also observed numerous copy number losses involving 89 cytobands (~37% overlap, <−1 threshold) in amplified and non-amplified samples (P1 vs. MDA1: Pearson’s r^2^ = 0.54, *p* < 0.0001, two-tailed; P2 vs. MDA2: Pearson’s r^2^ = 0.56, *p* < 0.0001, two-tailed) (Figure 1c).

Next, we assessed the ability of this approach in identifying somatic SNVs, copy number alterations (CNAs) and structural variants (SVs) in WGA samples from Parsortix harvested spike-in cells (S1 and S2) in comparison to bulk cancer cells (P1 and P2). The frequencies of coding variants in spike-in samples were similar to those of amplified and non-amplified bulk cancer cells. We found no significant differences in the frequencies of SNVs (p-0.7), indels (p-0.9), and substitutions (p-0.9) within technical replicates (Figure 1b). Among frequently mutated 40 genes, 8/40 showed overlap in in WGA (spike-in samples and bulk cancer cells) and non-WGA samples (Figure 1d). The mutational signature analysis revealed a similar substitution characterized by C > T transition as reported in COSMIC for MDA-MB-231 cells (Figure 1e). However, we observed very little concordance between the total number of coding variants (~8%) and genes (~11%) between amplified spike-in samples and bulk cancer cells (Figure 1f). Furthermore, these samples showed very little overlap for copy number gains (~5%) and losses (~7%) (Figure 1g). For SVs, no overlap of somatic SVs and genes were found between amplified bulk cancer and spike-in samples. One of the possible explanations for the discrepancy in the overlap of somatic alterations may be due to cellular heterogeneity within the same type of cancer cells [30,31]. Despite the importance of cellular heterogeneity, another reason may be due to the high variability of cell size. For spike-in samples, the genomic alterations were identified from a small subset of cancer cell populations with a large diameter (10 µm). However, bulk cancer cells have a broad distribution of size, including some cells that are smaller than WBCs and some that are larger than 10 µm in size. 

We have also evaluated the Parsortix efficiency for priming and cell capture rate by spike-in MDA-MB-231 cells (*n* = 100) in a healthy donor blood sample and processed with four different size cassettes—4.5 µm, 6.5 µm, 8.5 µm, and 10 µm. The Parsortix cell capture efficiency for 10 µm cassette is 68%. (Supplementary Figure S1a). In addition, the 10 µm cassette showed better priming efficiency than 4.5 µm, 6.5 µm, and 8 µm with no air bubbles (Supplementary Figure S1b). This shows that spike-in samples can be purified from blood; however, there is a variation of cell capture rate in the different sizes of cassettes. Studies have found that CTCs are significantly larger than other cells contained in peripheral blood [32]. It is also known that CTC size can be even smaller than or similar to leukocyte size [33,34]. We used a 10 µm cassette in this study in order to minimize the background leukocytes. However, we believe that size overlap between CTCs and leukocytes could be also one of the reasons, to some extent, for the observed discordance between spike-in samples and pure cancer cells in addition to heterogeneity. 

These cell line spike-in experimental results demonstrated that our integrated approach yielded sequencing libraries of high fidelity from bulk and Parsortix isolated cancer cells for WES. The combination of the Medexome target enrichment approach with NGS technology performed well on low-quantity samples by providing consistency and better uniformity of coverage. With technical replicates, the results of the workflow were reproducible and showed the margin of error based on the high throughput approach. This workflow was also effective both in terms of performance and accuracy of the results for WGA samples. These experimental results revealed that this methodology helped identify known mutational hotspots as reported for MDA-MB-231 within COSMIC, the Cancer Cell Line project, enabling an accurate variant calling using WES. In addition, this methodology helped identify novel mutation hotspots, copy number events, and structural variants and establishes the analytical features of specificity and accuracy in low-quantity and bulk cancer cells. Overall, these cell line spike-in experimental results provide methodologic validation and form the basis for in-depth genomic analysis of patient CTC samples.

### 2.2. The Landscape of Somatic SNVs in CTCs and Metastases

We have previously identified the variants from whole transcriptome sequencing in Parsortix isolated CTCs and fresh tissue from MBC patients [35]. In the current report, we evaluated the ability of the WES Medexome assay to detect variants in Parsortix isolated CTCs and matched FFPE tissue samples. FFPE tumor tissue was available from 4/5 MBC patients’ metastatic biopsies. WBCs from the same patients were profiled to eliminate germline contaminants. 

**Figure 2:**
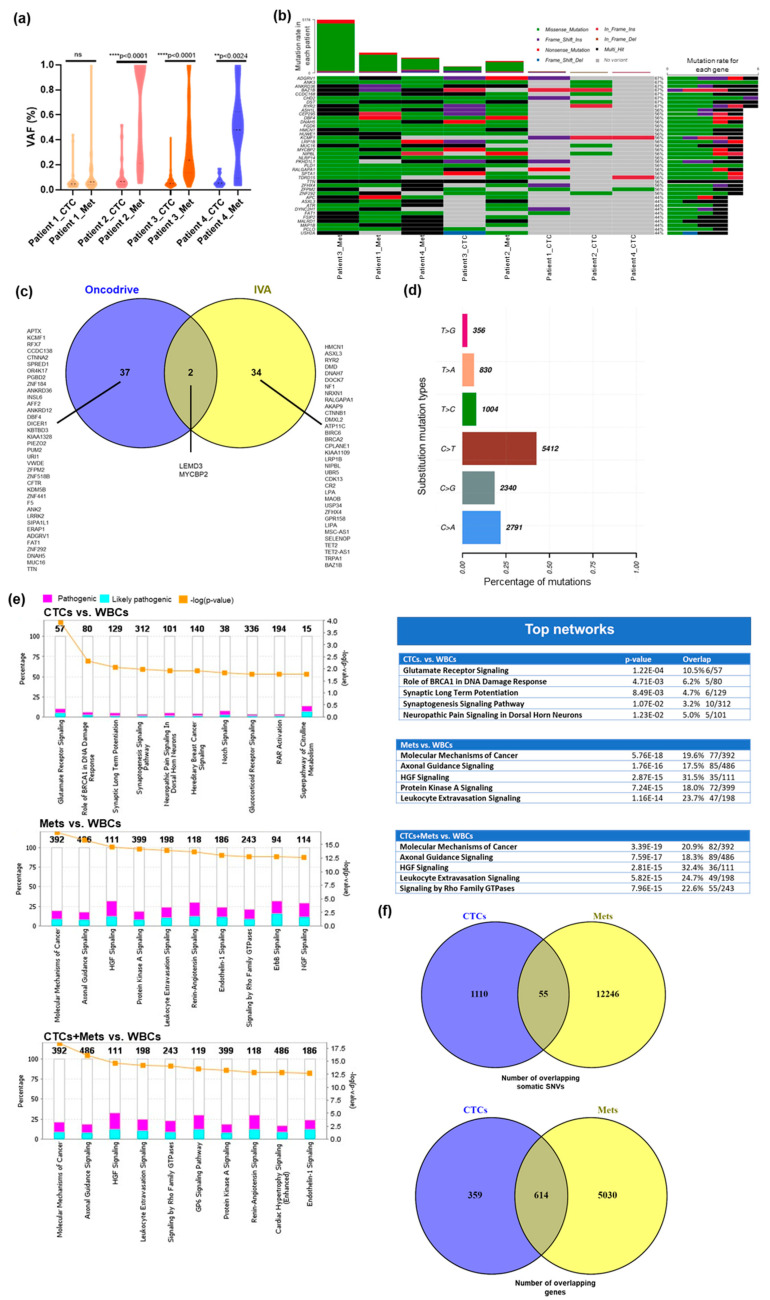
Overview of somatic mutations in circulating tumor cells (CTCs) and matched metastatic tissues (**a**) Violin plot showing the distribution of VAFs across four CTC samples with matched formalin-fixed paraffin-embedded (FFPE) tissue (two-tailed paired t-test, *p* < 0.05). The black dots indicate the median and quartile of VAFs and violins extended to represent the entire data range. (**b**) Oncoplot showing the 40 most frequently mutated genes color-coded by type of mutations in CTCs and matched metastatic tissues. The percentage to the right of the oncoplot shows the percent of samples with variants for the corresponding gene. Right, barplot shows the mutation rate in each of the 40 mutated genes; Top, barplot shows the mutation rate for each patient for 40 most frequently mutated genes. By default, samples are ordered by the most frequently mutated genes. (**c**) Overlap of the most frequently mutated genes identified by Maftools and IVA to identify shared cancer drivers in CTCs and metastases. (**d**) Distribution of base substitutions in CTCs and metastases revealing a signature characterized predominantly by C > T followed by C > A substitutions. The percentage of mutations are shown on the x-axis and substitution mutation types are on the y-axis. (**e**) Bar chart showing the enriched canonical pathways in CTCs vs. white blood cells (WBCs), Metastases vs. WBCs, and CTCs + Metastases vs. WBCs. The y-axis on the left shows the percentage of genes overlapping in each pathway having pathogenic (pink) and likely-pathogenic (blue) variants. The y-axis on the right shows the significance level. The number on the top of each stacked bar indicates the total number of genes present in each pathway. The orange line represents the threshold value (0.05) for the significance level for -log(*p*-value)). The graph is displaying only those entities that have a -log(*p*-value) greater than 1.3. (**f**) The number of overlapping somatic SNVs and genes in CTCs and metastases.

The Medexome capture assay allowed us to identify variants in 5/5 (100%) CTCs and 4/4 (100%) FFPE tumor samples. After excluding noncoding variants, 12,311 variants were identified in FFPE samples, spanning 5644 genes, including 11,527 SNVs, 579 indels, and 205 substitutions. Among CTCs samples, a total of 1166 somatic coding variants were identified in 972 genes, including 783 SNVs, 352 indels and, 31 substitutions. The percentage of reads on target was higher in CTCs samples than FFPE samples. These findings indicate that target capture efficiency is affected by low quality DNA. We observed a low percentage of on-target reads in WBC samples, however, these samples achieved ≥15x–18x coverage over targeted coding regions. In addition, the average Phred-based quality scores for variants in CTC, FFPE, and WBC samples were above threshold ≥30 (Table 2). Of note, we found that the overall VAF distribution of somatic SNVs in CTCs was lower than the FFPE tumor, which reached statistical significance for three of the samples (patient #2 and #3: *p* < 0.0001; patient #4: *p* < 0.0024) (Figure 2a), suggesting low tumor purity in CTCs, as is expected given a background of abundant leukocytes. Identification of low VAFs may also become particularly challenging in low-quality and low-quantity samples, such as CTCs and FFPE, with a limited amount of DNA that may not contain enough molecules contributed by the tumor genome. Lower VAFs have been detected in liquid biopsies compared to tumor biopsies and deep sequencing is often required to detect low abundance variants accurately [36,37,38]. Here, we used the hybridization-based target enrichment method that provides more uniform coverage and higher sensitivity in detecting low and high VAF [39,40,41]. For the identification of known and novel variants that are present at low frequencies in CTCs, we first screened them against dbSNP and COSMIC databases. We then applied two in silico prediction methods SIFT [42] and Polyphen [43] for predicting the functional consequence of the low-frequency variants [44]. Interestingly, CTC samples harbored alterations in 27 FoundationOne and 33 MSK-IMPACT actionable genes. In these five evaluable CTC samples, we found that 3/5 (patients # 1, 2 and 3) samples harbored shared mutations in potentially actionable genes (ARID1A, CHD2, NCOA3, and CSF3R). We also identified 87 actionable genes that showed overlap with FoundationOne and 109 genes with MSK-IMPACT in FFPE samples. Only 24 actionable genes were found to be in common in CTCs and matched FFPE metastatic tissue biopsies in 2/4 patients analyzed (Table 3). The OncoKB database was also used to evaluate the level of clinical evidence of detected mutations. We identified only two actionable hotspots in metastases (C420R in patient #1 and E542K in patient #3) in the PIK3CA gene that has supported clinical evidence (level 1 and 3). Interestingly, the FoundationOne results for patient #1 also showed the presence of the PIK3CA C420R hotspot mutation in metastatic tissue.

We next applied the Oncodrive function of Maftools to identify driver genes in which a mutation occurs more frequently in CTCs and metastatic tissues than expected by chance, taking into consideration the gene mutation rate and sample mutation burden. The analysis identified 39 statistically significant genes with an FDR cutoff of 0.05. The *p*-values and FDR for the 39 genes are listed in Supplementary Table S1. Indeed, 2/5 CTC samples (patients 2 and 3) showed at least one mutation in 6/40 of the most frequently mutated genes and were also identified as tissue specific. Patient #3 also harbors four multi-hit genes (ANKRD36, CCDC168, MUC16, and TTN) that were mutated more than once in both CTCs and metastases (Figure 2b). Oncodrive is limited by a bias favoring the detection of oncogenes and is less efficient in identifying randomly distributed mutations in tumor suppressor genes [45]. We also used Ingenuity Variant Analysis (IVA) to identify driver genes that are mutated in at least 4/9 cases (>40%) in both CTCs and metastases. Both of these approaches identified driver gene mutations in breast cancer-associated genes, such as MUC16, NF1, and BRCA2, suggesting that using different predictive computational tools improves the sensitivity and specificity in identifying cancer somatic mutations (Figure 2c). We next performed mutational signature analysis using Maftools. Across all 6 substitution types, a higher prevalence of the three most frequent substitutions (C > T, C > A and C > G) was identified in CTCs and metastases (Figure 2d). These signatures are also thought to be responsible for most of the mutations in 21 primary breast cancer genomes [46] and are also identified as a dominant mutation type in ER-positive breast cancer patients [47]. Nonetheless, the total number of coding variants and gene overlap was found to be very low in CTCs and metastases (Figure 2f) highlighting the intra-patient heterogeneity of CTCs versus paired metastasis.

We identified biological pathways that were altered by coding mutations and indels in CTCs and metastases. The canonical pathway module of IPA identified 21 signaling pathways in CTCs, 324 in metastases and 410 in CTCs and metastases taken together (CTCs + metastases) that have a -log(*p*-value) greater than 1.3. We defined CTCs + metastases as a group in which we analyzed all clinical samples (5 CTCs and 4 metastases) as one group. Of note, we found 16 overlapping pathways in CTCs and metastases primarily involved in the role of BRCA1 in DNA damage response, hereditary breast cancer signaling, ATM signaling, and several others (Figure 2e). The Fisher’s exact test was used to identify the most common significant pathways (*p* < 0.05). 

These results demonstrate the potential of size-based Parsortix technology in variant analysis to provide insights into CTCs genomic instability. The average quality score of CTC, metastases, and WBC samples was almost similar, and thus made it possible to analyze somatic variants, CNAs, and SVs in these specimens. The discordance in somatic alterations was found in all patients between CTCs and tissue metastases, revealing inter- and intra-patient heterogeneity in MBC. These results showed the feasibility of the Medexome assay in capturing cellular heterogeneity. Overall, these results showed that WES of low-quantity and low-quality samples could capture the landscape of somatic alterations as well as identify signaling pathways and provide a comprehensive profile of tumor heterogeneity in MBC. 

### 2.3. The Landscape of Somatic SVs in CTCs and Metastases

**Figure 3:**
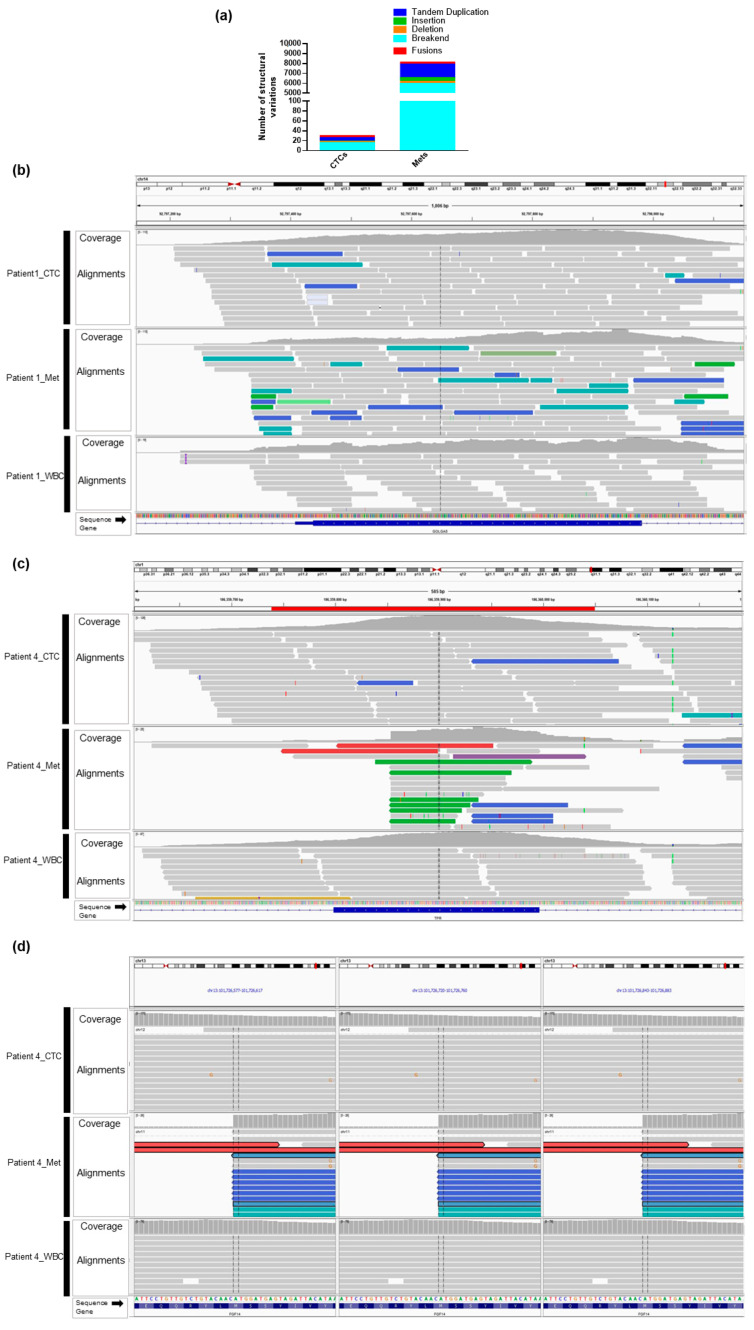
Overview of somatic structural variants (SVs) in CTCs and matched metastatic tissues. (**a**) Distribution of somatic SVs in CTCs and metastatic tissue depicting a high proportion of translocation breakends and tandem duplications. (**b**) Screenshot from IGV showing the coverage and read alignments (gray) in CTCs, metastases, and WBCs of patient #1 and inversions in colored bars (teal and blue) in the GOLGA5 gene. There is no inversion in the matched WBC sample. (**c**) Screenshot from IGV showing the coverage and read alignments (gray) in CTCs, metastases, and WBCs of patient #4 and duplications (green) in the TPR gene. There is no duplication in the matched WBC sample. (**d**) Split-screen view from IGV showing the translocation breakpoint in the FGF14 gene on chromosome 13 in patient #4 metastases. There is no translocation breakpoint in CTC and WBC samples.

An increase in genomic instability has been linked to a concomitant increase in the frequency of structural rearrangements or fusions [48,49]. Among SVs, the chromosome rearrangements are more frequent in breast cancer. However, the frequency of these variations is currently unknown as these alterations are not easily detected in low quantity and low-quality samples with standard techniques such as array comparative genomic hybridization (CGH) or fluorescence in-situ hybridization (FISH). Here, we examined the distribution of SV signatures (larger mutations, 50 bp or larger) in breast cancer CTC and FFPE specimens. A total of 30 somatic coding SVs in CTCs and 8117 SVs in metastases were detected using the Manta algorithm. The number of SVs and the relative distribution between SV classes varied considerably in CTCs and metastases (Figure 3a). The majority of somatic SVs were intrachromosomal although a few affected different chromosomes. 

Across CTCs and metastases, the breakpoints of 16 rearrangements in CTCs and 5771 in metastases were identified. It is important to note that rearrangements not only create novel driver oncogenes but can also disable tumor suppressors. It has been shown that ~16% of the osteosarcomas, which lacked commonly hotspot TP53 mutations, were instead reported to have recurrent rearrangements in intron 1 of TP53 [50]. We also identified SV rearrangements in known oncogenes (GOLGA5, MET, MYB, NCOA4, PIK3CA, PTPN11, SS18, TFG, and TPR) tumor suppressors (APC, ARGEF12, ATM, BRCA1, BRCA2, CDH11, FBXW7, JAK2, MAP2K4, MLH1, MSH2, FGF14, CDC73, and NF1) in CTCs and metastatic samples. Figure 3b–d shows the three examples of structural variations in patients #1 and #4.

Gene fusions represent a unique form of genetic alterations and have been used as a diagnostic marker for many cancers. Our analysis revealed a total of 4 gene fusions in CTCs and 207 in metastases in patients #1 and #4. We also queried for gene fusions that occurred in a single case (singleton fusions) or more than one case (recurrent fusions) in CTCs and metastases. For some of the singleton fusions, one of the involved genes was among five clinically actionable genes NOTCH2, NF1, ATM, CDC73, and KMT2C (Supplementary Table S2).

Tandem duplications and deletions were also the commonly observed rearrangements in breast cancer. Interestingly, we found the majority of larger duplications (>1 Kb) in CTCs and smaller duplications (~1 Kb) in metastases. Small size deletions (~1 Kb) were detected in exonic regions in both CTCs and metastases. Notably, we could not find any overlap of specific chromosomal rearrangements between CTCs and metastases when comparing the pairwise genomic coordinates for both ends of the structural variation. 

### 2.4. The Landscape of Somatic CNAs in CTCs and Metastases

**Figure 4:**
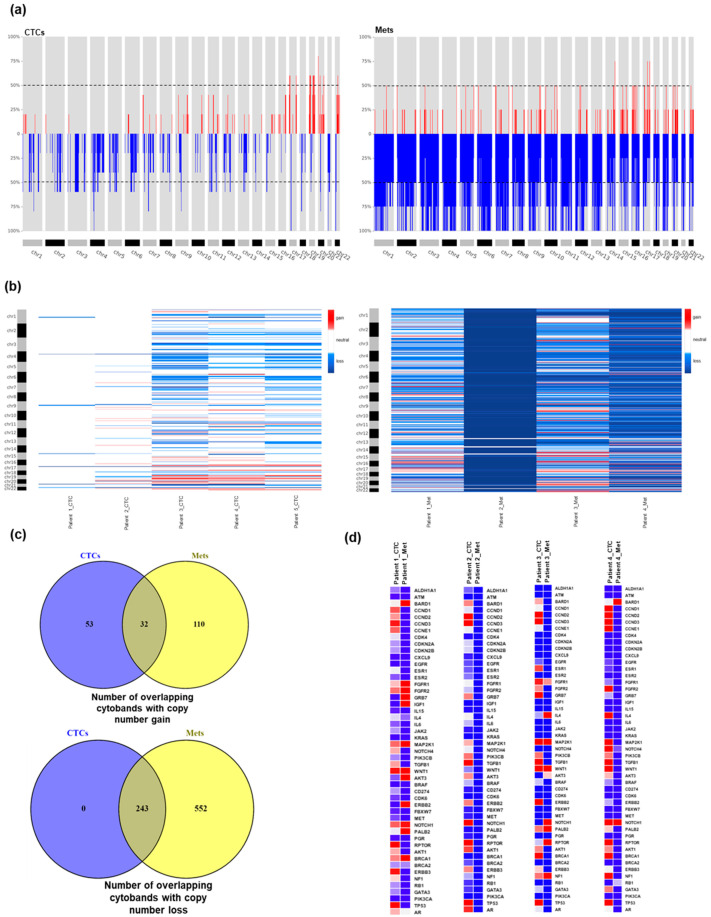
Overview of copy number alterations (CNAs) in CTCs and matched metastatic tissues (**a**) Overall copy number landscape showing the high prevalence of focal alterations in all CTCs and metastases. The y-axis shows the frequencies of the occurrence of somatic gains (red) and losses (blue) in sub-cytoband regions. (**b**) Heatmap showing the segmented copy number profiles at chromosomal arm level in each of the samples in CTCs and metastases (gain = red, loss = blue, and neutral = white). (**c**) The number of overlapping cytobands depicting copy number gain and loss in CTCs and metastases. (**d**) The heatmap showing the gains and losses in 49 potentially actionable genes in CTCs and metastases.

Since specific cytogenetic abnormalities are dominant features in breast cancer, we further evaluated somatic CNAs in paired samples at the sub-cytoband level. We identified a total of 7910 CNAs (3994 amplifications and 3916 losses) in CTCs and 41,154 (11,970 amplifications and 29,184 losses) in metastases. The re-segmentation approach was applied using CNApp [51] that adjusts for technical variability due to sample noise and corrects for the estimate of tumor purity. Re-segmented data was used to calculate the broad (BCS), focal (FCS), and global (GCS) CNA scores. Across the entire genome in CTCs and metastases, the most prevalent CNAs were focal events (affecting less than 50% of the chromosome arms) that occurred at a higher frequency than broad aberrations (affecting more than 50% or whole chromosome arms) (Figure 4a). At the sub-cytoband level, we found an excess of deletions rather than amplifications in all four paired samples (Figure 4b). Interestingly, the overlap rate of CN events (gain and loss) in CTCs and metastases was higher than SNVs and SVs. CTCs and metastases harbored an overlap of 22.5% gain and 30.6% loss at sub-cytoband levels (Figure 4c). We next compared the CNAs in CTCs and metastases in our identified potentially clinically actionable breast cancer targets [52]. Copy number alterations showed that CTC samples contained the most frequently altered sub-cytobands including gains in proliferation markers (CCND1, CCND2, CCND3, RPTOR, and CCNE1) and stem cell signaling targets (TGFB1, TBXA2R, and WNT1). The loss in copy number includes immunological markers (IL12A, IL15, and IL6) and DNA repair targets (ATM and BRCA2) in CTCs. Of note, deletions at 13q (BRCA2 and RB1) commonly identified in breast cancer were discovered in 3/5 (60%) CTC samples (Figure 4d). We found significant reproducibility of the copy number gain and loss patterns between CTCs and metastasis for 41/49 actionable genes. The percentages, p- and q-values are shown in Supplementary Table S3. We also found significant variability in CTCs and metastases in the frequencies of CNA calls for some actionable genes, for instance, TP53 deletion in metastases and amplification in CTCs. We also observed the reproducibility of copy number patterns at a global level in CTCs and metastatic tissues but to a lesser extent. 

The results of CNA analysis showed that focal regions, including well-known oncogenes or tumor suppressor genes, are frequently amplified and deleted in CTCs and metastases. These results also demonstrate the ability of the Medexome assay to identify potentially actionable copy number events from clinical samples. CNA patterns at certain chromosomal loci were consistent between CTCs and metastases. We also observed substantial discordant CNAs between CTCs and corresponding tissues, suggesting diverse clonality and tumor heterogeneity between patients. Taken together, this analysis suggests that a single-site biopsy contains only a minority of genomic alterations present in MBC.

## 3. Discussion

This study demonstrates that it is possible to generate WES data from low-quantity and low-quality samples. We combined the WGA method with a hybridization capture target enrichment approach for exome sequencing of CTCs isolated via the microfluidic device and matched FFPE samples from MBC patients. We used the microfluidic ANGLE Parsortix technology that captures CTCs based on the physical differences of cell size and deformability with an efficiency of 50–60% while depleting leukocytes 10^6^ -fold down to 200–800 WBCs per mL of blood [53,54]. The low amplification bias MDA technology that generates long DNA fragments (>10 kb) was used to amplify DNA from experimental and clinical samples. This methodology achieved exome coverage greater than 55% across all experimental, CTC, and metastases samples and thus made it possible to do a comprehensive analysis of the coding regions having SNVs, CNAs, and SVs.

Comparing the concordance of somatic SNVs across CTCs and metastases, we identified an overlap of only 55 exonic variants and 614 genes using the WES approach in MBC patients. A stringent selection criterion was applied for SNV identification. Matched WBCs were also assessed in parallel for each of the patients for the identification of germline single nucleotide polymorphisms (SNPs) and to control for clonal hematopoiesis. We found many somatic variants in metastases such as PIK3CA p.C420R in patient #1 and NF1 in patient #2 that were not present in matched CTCs. Likewise, CTC samples also harbored many somatic alterations that were not present in metastases. Many studies based on single cells and clusters have shown that CTCs have a high level of heterogeneity in terms of their mutational and transcriptional landscape [2,55,56,57,58,59,60,61]. Importantly, we identified 51 actionable genes in CTCs and metastases that also showed overlap with FoundationOne, OncoKB, and MSK-IMPACT gene panels (Supplementary Figure S2). Additional findings that are noteworthy are that we identified the 39 most frequently mutated genes in CTCs and metastases, and some of them such as MUC16, ANKRD36, and DNAH5 are known to be biologically important in breast cancer. We cannot exclude the possibility that some of these mutations were CTCs-only or metastases-only or could be due to heterogeneity. However, these alterations could help us understand the mechanisms that underlie metastatic spread. In metastatic prostate cancer, Lohr et al. (2014) reported an overlap of 51% of the somatic SNVs between CTCs and metastases using WES [62]. Paoletti et al. (2018) observed 85% concordance between somatic alterations in single and pooled CTCs subjected to a targeted sequencing approach for a panel of 130 genes and corresponding tissue metastases profiled by WES in breast cancer [63]. Notably, both of these studies evaluated the concordance of somatic alterations in individual CTCs vs. metastatic biopsy, suggesting that single CTCs exhibit high genomic concordance to metastatic tissue. However, in our study using WES with maximal coverage of 4600 medically relevant genes, we identified only a minor proportion of shared somatic SNVs (0.4%) and genes (11%) between CTCs and metastases. The Medexome panel with enhanced coverage of 4600 genes has inherently richer data than targeted sequencing but we observed a lower percentage of variants than a list of genes selected a priori relevant to a specific disease. These limitations could be contributed by the heterogeneous nature of WES in terms of uneven coverage along the length of exons, which affects variant calling analysis. For instance, a study report of Wang et al. (2017) revealed that some exonic regions are captured poorly even at a high average read depth of >75x, which may result in missed variant calls [64]. In many cancer studies, WES has been used as an initial discovery tool for identifying significantly mutated genes. These genes are further validated via a targeted sequencing approach at a much higher sequencing depth than WES, as done by Paoletti and colleagues [63]. Although, targeted sequencing enables the identification of rare variants at a higher depth than WES. However, it is less efficient for the identification of structural variants and copy number events. For example, in our recent study, we observed that SNP array platforms identify copy number changes to a reasonable degree of accuracy than targeted sequencing [52]. For discordant genomic alterations between CTCs and metastastis, there are at least three other possible explanations of this discrepancy. First, a single-site metastatic lesion was unable to capture the global repertoire of the somatic mutational landscape [65]. Second, these CTCs are derived from different clones of heterogeneous distant metastases. Third, leukocyte contamination in CTCs and tumor stroma in metastatic lesion tumors may also confound variant identification. In addition, discordant somatic alterations and gene expression profiles in CTCs and metastasis may reflect the clonal evolution caused by therapy pressure [61,63,66,67]. Whole-genome analysis of multiple biopsies in metastatic triple negative breast cancer (TNBC) patients also revealed extensive spatial and temporal heterogeneity in SNVs, CNAs, SVs, and polymorphisms, suggesting the in-depth genomic analysis of multiple biopsies for each patient [68]. Moreover, intra-tumor heterogeneity has also been observed by whole-exome multi-region spatial sequencing between primary/metastatic tumors, with 63% to 69% of somatic mutations not detected in all tumor specimens when performing WES of multiple different regions of the same tumor [65].

Multiple somatic SVs are often found in breast cancer genomes. Tandem duplications, the most important SV class, are known to produce oncogenic fusion genes in cancers. We found 23% duplications in CTCs and 17% in metastatic tissues. The interpretation of tandem duplications has not been specifically addressed in the current American College of Medical Genetics and Genomics (ACMG)/Association for Molecular Pathology (AMP) guidelines. However, Richardson et al. [69] suggest that their high prevalence is associated with the presence of defects in DNA repair. Interestingly, we also identified many DNA repair genes such as BRCA1, MSH3, MLH1, and ERCC3 having tandem duplications in only metastases. For SNV analysis, we identified 16 overlapping pathways in CTCs and metastases involved in the role of BRCA1 in DNA damage response, hereditary breast cancer signaling, and ATM signaling. These results suggest that CTCs could survive a potentially lethal dose of chemotherapy and make the cancer cells resistant to DNA-damaging therapies. Our data shows that intra-chromosomal rearrangements are most prevalent in CTCs and metastases. Gene fusions are the result of chromosomal rearrangements and translocations. We identified gene fusions in CTCs in patient #1 and in metastases in patients #1 and #4. Two of the intrachromosomal gene fusions in ASPM1 and TRPS1 genes were also found to be recurrent. Moreover, for singleton fusions identified, one of the involved genes from two genes’ combination is potentially breast cancer related actionable genes (Supplementary Table S1). We observed no overlap of SSVs in CTCs and metastases which revealed substantial inter- and intrapatient molecular heterogeneity. 

The CNAs profile generated from CTCs and metastases showed a higher prevalence of alterations in focal rather than broad chromosomal arms. Interestingly, we observed extensive copy number loss rather than gains in CTCs and tumor tissues. Our approach facilitates the identification of deletions of chr13 and 16q, in all CTCs and metastases, commonly found aberrations in breast cancer. We also found low-level gains in chr6p21, 23, and 25 cytobands in CTCs. Interestingly, the gain in chr6p elevated HLA expression profiles and suppressed natural killer (NK) cell activation [70]. When comparing the copy number profiles of CTCs to metastases, approximately a quarter (22.5% gain and 30.6% loss) in CTCs and metastases of sub-cytobands genomic regions showed similarity for CNAs. These findings provide a strong rationale for exploring copy number changes for potential clinically actionable genes that are linked to food and drug administration (FDA)-approved or investigational therapeutics. Of note, we found high similarity in copy number profiles of many potentially clinically actionable genes in CTCs and metastases. 

Many studies have shown that WGS is more comprehensive and powerful than WES in detecting exome variants [71,72,73]. The major challenge in WES is the target coverage uniformity with regard to capturing a reasonable number of reads mapped to the target regions. The estimates provided by the previous studies for the depth of sequencing coverage for WES are variable. Meynert et al. (2014) recommended the mean on-target read depth of 17x–37x for exome sequencing, 13x local read depth for alleles and heterozygous SNVs, and 3x for homozygous SNV identification [74]. Clark et al. (2011) estimated that the common threshold of 10x is required to detect >90% of the targeted bases. The authors also show a strong inclination for a target enrichment approach in comparison to WGS because higher base coverage after enrichment identifies variants that are missed by WGS [75]. We performed WES at 100x coverage and assessed the percentage of targeted bases covered at depths of at least 1x and 20x. We found high coverage in experimental samples and clinical samples. Nonetheless, in WBC samples, the percentage of target bases was less but the data analysis was restricted to target reads with a Phred-based quality score >30. Since our results are not fully consistent with the previous studies, we also did a side by comparison of sequencing results and quality statistics with a pilot study on NGS of paired CTCs, FFPE tumors, and peripheral blood mononuclear cell (PBMC) samples in hepatocellular carcinoma [76]. Although, both of the studies have variation in terms of NGS platforms and variant pipelines. However, the comparison between both of the studies showed that NGS libraries generated from CTCs and FFPE samples are somewhat similar in the quality of statistics of the sequencing data (Supplementary Table S4). 

We present a systemic workflow by combining a microfluidic-based CTC capture approach with WES to identify the complete repertoire of genomic alterations in MBC. The major limitation of our study is that a sample size of five breast cancer samples with single-site biopsies from only four patients is an insufficient cohort to generalize to all breast cancer patients and subtypes. However, this study was performed as a pilot study and the central aim was to assess the technical feasibility of integrating WGA with WES in low-quantity and low-quality samples. Each patient served as their own control, which is appropriate given that breast cancer is a heterogenous disease. Given that there are five major intrinsic subtypes of breast cancer, we were careful to avoid generalizing our findings in the context of any subset analysis for intrinsic subtyping. Many studies based on the sequencing of CTCs have enrolled a few samples and analyzed single CTCs [21,77,78]. Lohr et al. (2014), in their study, have enrolled 34 metastatic prostate cancer patients [62]. However, the WES was performed only on two patients because only two subjects yielded a sufficient number of CTCs. In comparison to these studies, our study has performed WES on a larger number of samples and is not based on single CTCs. Moreover, the Medexome assay we used is based on WES with enhanced coverage of 4600 genes that has inherently richer data than the sequencing assays used in previous studies. In addition, this workflow was tested on different samples from each patient, assaying CTCs, tumor tissues, and WBCs. Nonetheless, the approach performed well, with experimental and clinical samples collected in Streck tubes, and demonstrated the technical feasibility of WES of CTCs. The workflow showed compatibility when combining the WGA of CTCs and FFPE tissues with hybridization capture technology. Although the sample size was small, it represents an example of the optimization of the WES procedure in experimental samples and validation in clinical samples. Indeed, our results highlight the substantial intra-tumor heterogeneity suggesting that N-of-One designs are essential to implement personalized medicine since each tumor has unique biology and each patient served as their own control in an omics based analysis that evaluated many biomarkers in a small patient cohort [79]. Of note, our data represent a proof-of-principal demonstrating the possibility for our integrated experimental design for low input DNA and opens the door to applying the WES approach in rare CTCs and FFPE samples. Moreover, we have used bioinformatics tools with stringent statistical filters to identify true somatic variants. We have identified variants in all five CTCs and four FFPE samples. The presence of somatic variants was also verified with COSMIC, Human Gene Mutation Database (HGMD) and ClinVar. The identification of variants in all of the five patients demonstrates that the strategy is viable and WES fulfills the analytical features of specificity and accuracy required for low-quantity and low-quality samples. The VAFs in FFPE samples were higher than in CTCs, suggesting a higher tumor content in tissues uncovers a larger number of SNVs, SVs, and CNAs than CTCs. We identified many known and unknown variants; however, high discordance in somatic alterations was found between CTCs and matched FFPE samples. It is unlikely that discordance was due to WGA and sequencing as MDA technology has an extremely low error rate of 10^−5^ [18,80]. Interestingly, Razavi and colleagues identified only 24.4% of somatic mutations in plasma DNA in matched tumors, suggesting the contribution of clonal hematopoiesis to molecular discordance [81]. The authors also revealed that >50% of somatic mutations in cell-free DNA (cfDNA) in cancer patients originated from WBCs and were attributable to clonal hematopoiesis.

Our study has several strengths. We utilized healthy donor blood samples and spike-in MDA-MB-231 breast cancer cells to more closely mimic the CTCs from patient samples. We included matched WBC gDNA and performed sequencing at the same coverage (100x) used for CTCs and FFPE samples to control for the contribution of WBC somatic mutations, avoiding the detection of clonal hematopoiesis. We used Parsortix technology capable of capturing heterogeneous CTC subpopulations expressing epithelial, mesenchymal, and stemness markers. Many studies have also shown that CTCs have a high degree of heterogeneity [53,61,63]. Such an analysis of heterogeneity is often difficult and presents a serious challenge to variant calling and is critical for precision medicine. This would imply that more CTC samples and multiple biopsies would be needed to understand the full extent of genomic diversity in metastatic cancers. Recently, a joint review from the American Society of Clinical Oncology (ASCO) and the College of American Pathologists (CAP) provided an assessment on clinical ctDNA assays and a framework to guide future research [82]. However, clinical implementation of WES in liquid or solid biopsies has been much slower due to a lack of robustness of preanalytical and post analytical experiments and clinical validation studies. 

Our results highlight the substantial differences in somatic alterations between CTCs and their corresponding tissue metastasis, suggesting that a single metastatic biopsy’s results may be markedly different from that of the CTCs. Thus, more caution should be exercised in selecting genes and variants that allow for the complete genomic analysis of CTCs to provide insights into the biology of MBC. As such, an understanding of tumor heterogeneity is important, as most of the mutations might be found in one cell and not another, and that could be the main cause of therapeutic failure. 

In conclusion, our discovery approach facilitates a deeper understanding of CTC biology and supports the feasibility of whole-exome sequencing of low-input CTCs and low-quality FFPE material in MBC patients. The molecular discordance between CTCs and metastatic tissue identified in our MBC study may have potential clinical significance as this data reveals many actionable alterations only present either in CTCs or tissue metastasis. These results suggest that the identification of potential actionable therapeutic targets may be missed when relying on a single-site biopsy of metastatic lesions. The results presented provide an insight into the potential clinical utility of the WES workflow in MBC and also emphasize the need for validating this assay in other disease settings. The multiple metastatic lesions may improve outcomes by providing accurate interpretation and the full spectrum of somatic mutations, thus facilitating more effective treatment strategies for individual patients.

## 4. Materials and Methods

### 4.1. Cell Culture and Spike-in Experiment

The human breast cancer cell line MDA-MB-231 cell line was purchased from ATCC and cells were maintained in 5% CO_2_ at 37 °C incubation in complete Dulbecco’s Modified Eagle Medium (DMEM) medium (Life Technologies, Carlsbad, CA, USA). Cells were washed twice with 1xPBS (Life Technologies, Carlsbad CA, USA). At 75–80% confluency cells were detached using trypsin-EDTA (0.05%) (Life Technologies, CA, Carlsbad, USA). For the spike-in experiment, MDA-MB-231 cells (*n* = 50) were spiked into 7.5 mL healthy donor whole blood in a Cell-Free DNA BCT tube (Streck, La Vista, NE, USA). MDA-MB-231 bulk cancer cells were used as a positive control. We used WBCs isolated from peripheral blood not processed on the Parsortix as a negative control (no spiked cells). The spike-in experiment was done in duplicate.

### 4.2. Patient Population

Blood samples (7.5 mL for CTCs and 2 mL for WBCs isolation) were collected into Streck tubes (Streck, La Vista, NE, USA) from five eligible patients with MBC treated at the University of Southern California (USC) and Los Angeles County (LAC) medical center. The study protocol HS-15-00741 (Date of approval: 17 December 2019) was approved by the USC Institutional Review Board (IRB) Committee. The samples were collected after obtaining written consent in the HS-11-00208 protocol (Date of approval: 30 July 2019) approved by the USC IRB Committee. The metastatic FFPE-matched tumor samples were available only from four patients of the five eligible patients (prior to receipt of systemic treatment). For FFPE samples, we did not utilize laser capture dissection. However, the tumor cellularity was 30% for cut section curls from FFPE samples. The investigations were carried out following the rules of the Declaration of Helsinki of 1975 (Available Online: https://www.wma.net/what-we-do/medical-ethics/declaration-of-helsinki/), revised in 2013. The clinicopathological information of the patients is shown in Table 4.

### 4.3. CTC Capture and WBC Isolation

CTCs were enriched from whole blood using a Parsortix GEN3D10 Cell Separation Cassette (ANGLE plc, Surrey, United Kingdom) after 24hrs of blood draw, according to manufacturer’s instructions. After the CTC enrichment step and centrifugation, the cells were resuspended in 10 µL of PBS sc 1x (Qiagen, Germantown, MD, USA) and stored at −80 °C until further use. WBCs were isolated using the Ficoll-Paque density gradient centrifugation method. Briefly, 2 mL of blood of the blood sample was diluted with an equal amount of 1X HBSS buffer (VWR Life Sciences, Denver, CO, USA). Four mL of diluted blood was layered over 4mL of Histopaque (Sigma-Aldrich, Milwaukee, WI, USA) and centrifuged at 700 *g* for 25 min at room temperature without the brake applied. The WBCs interface was carefully removed by pipetting and stored at −20 °C until further use. 

### 4.4. Isolation and WGA of Genomic DNA from the Cell Line, WBCs, and FFPE Tissues

Genomic DNA from WBCs was isolated using the QIAamp DNA Blood Mini kit (Qiagen, Germantown, MD, USA). For bulk cancer cells, an AllPrep DNA/RNA/Protein kit (Qiagen, Germantown, MD, USA) was used as per the manufacturer’s instructions. The DNA extractions from FFPE samples were made from a 10 µm thick section using the GeneRead DNA FFPE kit (Qiagen, Germantown, MD, USA), as per the recommended instructions. The WGA of spike-in and clinical samples was conducted using the Repli-g Single cell kit (Qiagen, Germantown, MD, USA), according to the manufacturer’s protocol. For FFPE samples, the Repli-g FFPE kit (Qiagen Germantown, MD, USA) was used for WGA. All the DNA samples were quantified using a NanoDrop-2000 (ThermoFisher Scientific, Carlsbad, CA, USA) and Qubit 2.0 Fluorometer (ThermoFisher Scientific Inc., Waltham, MA, USA). WGA quality control was performed by Ampli1 QC kit (Menarini Silicon Biosystems, Huntingdon Valley, PA, USA) to check DNA integrity by conducting multiplex PCR of 4 targets at chromosome 12p, 5p, 17p, and 6p.

### 4.5. Pre-Capture Sample Processing

Library preparation was performed on a total of 22 samples (20 WGA samples (experimental samples: spike-in MDA-MB-231 samples (*n* = 2), positive control bulk MDA-MB-231 cells (*n* = 2) and negative control WBCs (*n* = 2); clinical samples: CTCs (*n* = 5), metastases (*n* = 4) and WBCs (*n* = 5)) and 2 non-amplified MDA-MB-231 samples (*n* = 2)). For each DNA sample, the barcoded library was prepared from 500ng of WGA material using the KAPA HyperPlus kit (KAPA Biosystems, Wilmington, MA, USA) as per the manufacturer’s guidelines. After enzymatic fragmentation, size distribution was checked using the Agilent2100 Bioanalyzer (Agilent Technologies, Santa Clara, CA, USA). The workflow contains basic steps for DNA library preparation including the end-repair of fragmented DNA, A-tailing, ligation of sequencing adapters, and amplification. The number of amplification cycles was also optimized to generate 1ug of amplified DNA. Library amplification was performed with 4 cycles. Barcoded libraries were purified using KAPA pure beads (KAPA Biosystems, Wilmington, MA, USA).

### 4.6. Hybridization Capture and Exome Enrichment

Exome capture was conducted with SeqCap EZ MedExome Target Enrichment Kit (Roche Diagnostics, Indianapolis, IN, USA) by pooling 4–6 libraries in equimolar concentrations. Pools were concentrated using KAPA pure beads (KAPA Biosystems, Wilmington, MA, USA). One thousand nanograms of purified library pool was then hybridized to the capture probes and the removal of non-hybridized molecules was carried out according to the manufacturer’s guidelines. Final libraries were quantified using the Qubit 2.0 Fluorometer (Thermo Fisher Scientific Inc., Waltham, MA, USA), and the fragment size distribution was determined with an Agilent Bioanalyzer 2100 (Agilent, Santa Clara, CA, USA). The libraries were then pooled equimolarly, and quantified via qPCR using the NEBNext Library Quant Kit for Illumina (New England BioLabs, Ipswich, MA, USA), according to the manufacturer’s instructions. Sequencing was done in 2x150 cycles format using Illumina HiSeq 2500 (in Rapid mode) and NextSeq 550 instruments. Library quality control and sequencing was performed at the USC Genome Core (University of Southern California, Los Angeles, CA, USA). 

### 4.7. Somatic Variant Calling and Interpretation

Raw sequencing reads (in the form of FASTQ files) were trimmed to remove potential adaptor sequencing using Trimmomatic [83]. The filtered reads were then processed following the Broad Institute’s best practices recommendation for variant calling (Available Online: https://gatk.broadinstitute.org/hc/en-us/articles/360035535912-Data-pre-processing-for-variant-discovery). In short, the reads were aligned to the human genome (hg38) using BWA-MEM [84] with default settings, and further processed using Gene Analysis Tool Kit (GATK) 4 to assign read groups, marking duplicates and base recalibration. The resulting processed BAM files were used to call short somatic variants (SNPs and Indels) using Mutect2 as explained in the Broad Institute’s Somatic Short Variant Discovery best practices workflow (Available Online: https://gatk.broadinstitute.org/hc/en-us/articles/360035894731-Somatic-short-variant-discovery-SNVs-Indels-), and the identified somatic variants were annotated using Annovar [85]. Annotated VCF files were also uploaded to IVA software (Qiagen, https://www.qiagen-bioinformatics.com/products/ingenuity-variant-analysis) to identify cancer-related mutations. The somatic variants were further analyzed by applying several filters. Our filtering cascade includes confident, common variant, predicted deleterious, genetic analysis, and cancer driver filters. In the confident filter we keep the variants with a read depth of at least 10. For common variant filter criteria, we exclude variants detected in 1000 Genomes project, Genome Aggregation Database (gnomAD), Exome Aggregation Consortium (ExAC), and NHLBI ESP exomes with an allele frequency of at least 3%. For the predicted deleterious filter, we keep only those variants that are pathogenic or disease-associated, according to HGMD and ClinVar. With the genetic analysis filter those variants are included that are associated with gain of function. We then applied cancer driver variant filters to keep variants that are associated with cellular processes and cancer related events/pathways, cancer therapeutic targets or published cancer studies, and found in COSMIC or The Cancer Genome Atlas (TCGA) databases at a frequency of 0.01%. Variants were also filtered if they occurred exclusively in intronic, intergenic, 3′UTR, or 5′UTR regions. 

The R package Maftools [86] was used to summarize the mutational spectrum and the variant classification distribution across samples, to identify the most frequently mutated genes and the frequency of each type of mutation, and to perform a cancer driver gene analysis using the Oncodrive tool [45] within Maftools. WES data are available at Sequencing Read Archive (SRA) under accession SUB7212646 (Bioproject: PRJNA644020).

### 4.8. Copy Number Alteration Calling and Interpretation

To estimate somatic CNAs, we used the GATK4 CNV workflow that detects copy number variants as well as allelic segments by normalizing the raw proportional coverage profile against a panel of normal (PoN) samples sequenced with the same capture technology. CNA was characterized by a measured copy number (expressed as log2 ratio), and by the extent of change in the genome. The CNA thresholds were determined according to the set of discrete copy number calls provided by GATK guidelines: amplification (+), deletion (-), and neutral (0). We used a re-segmentation approach using the CNApp tool to measure the burden of global, broad and focal CNAs in experimental and clinical samples [51]. The Fisher’s exact test was used to determine the concordance and discordance in the frequencies of actionable CNAs between CTCs and metastases.

### 4.9. Structural Variant Calling and Interpretation

SV calls were called from mapped data using Manta algorithm with default parameters in the Dragen Enrichment app in the Illumina basespace sequence hub. Manta detects deletions, inversions, tandem duplications, insertions, inter- and intra-chromosomal translocations, and gene fusions. Variants that passed all the set filters were further uploaded in IVA for functional interpretation with the same filtering cascade used for SNV analysis. The SVs were visualized and manually checked in Integrative Genomics Viewer (IGV). 

### 4.10. Ingenuity Pathway Analysis

Ingenuity Pathways Analysis (IPA) was used to identify key molecules and signaling pathways affected by genomic alterations in breast tumors. A list of genes having SNVs was subjected to IPA application (Qiagen, https://www.qiagen-bioinformatics.com/products/ingenuity-pathway-analysis). The variants that fit the American College of Medical Genetics (ACMG) criteria for classification as pathogenic or likely pathogenic were used to visualize the gene interactions [87].

### 4.11. Statistical Analysis

Statistical analysis for comparing the mutations and CNAs in amplified vs. non-amplified bulk cancer cells, amplified bulk cancer cells vs. spike-in samples, and CTCs vs. metastases was performed using GraphPad Prism 8.3.1 (GraphPad Software, San Diego, CA, USA). Correlation analysis of VAFs in cell lines between amplified and non-amplified samples was done using the Pearson r test. The Mann–Whitney test was used to evaluate the differences between the frequencies of coding variants. The two-tailed paired t-test was used to calculate the variability in VAFs for CTCs and metastases. The Fisher’s exact test was used to calculate the variability for the frequencies of CNAs. The two-stage linear step-up procedure of Benjamini, Kreiger, and Yekutieli by setting a false discovery rate (FDR) (Q) to 5% was used to correct p-values for multiple testing.

## Figures and Tables

**Table 1:** Summary of sequencing and alignment statistics across all experimental samples.

Sample ID	Total Reads Alignment (%)	On-Target Reads (%)	On-Target Bases that are Covered at Least 1x (%)	On-Target Bases that are Covered at Least 20x (%)	Coverage	Quality Score	GC (%)
MDA1 (Non-amplified gDNA) Replicate 1	99.9	74.1	99.6	98.9	105	33.4	47.9
MDA1 (Non-amplified gDNA) Replicate 2	99.9	71.2	99.6	98.8	102	33.6	47.6
P1 (Positive control- Amplified gDNA) Replicate 1	99.9	80.7	98.3	78.5	80	36.3	43.2
P2 (Positive control- Amplified gDNA) Replicate 2	99.9	79.6	98.5	84.9	132	36.1	43.1
S1 (Spike-in sample) Replicate 1	99.9	77.6	92.9	60.3	57	36.4	40.8
S2 (Spike-in sample) Replicate 2	99.9	78.5	94.1	63.2	61	36.4	41.1
WL1 (WBCs-Healthy control)-Replicate 1	99.9	75.0	93.2	70.3	70	36.3	40.2
WL2 (WBCs-Healthy control)-Replicate 2	99.9	76.4	88.7	54.7	55	36.6	40.2

**Table 2:** Summary of sequencing and alignment statistics across all clinical samples.

Patient	Total Reads Alignment (%)	On-Target Reads (%)	On-Target Bases that are Covered at Least 1x (%)	On-Target Bases that are Covered at Least 20x (%)	Coverage	Quality Score	GC (%)
Patient 1_CTC	99.9	73.1	99.1	79.3	142	38.2	42.0
Patient 1_Met	99.9	59.0	71.5	50.2	86	33.6	40.0
Patient 1_WBC	99.5	14.5	79.8	21.7	17	38.2	38.2
Patient 2_CTC	99.8	76.4	98.1	72.0	95	38.3	43.7
Patient 2_Met	99.9	64.0	43.8	13.4	72	32.0	45.0
Patient 2_WBC	99.9	13.0	73.4	18.5	15	38.4	38.3
Patient 3_CTC	98.3	71.5	97.0	72.8	82	38.0	43.8
Patient 3_Met	99.9	62.4	71.1	32.9	59	32.8	43.1
Patient 3_WBC	99.3	14.0	80.7	18.1	14	38.2	40.0
Patient 4_CTC	99.8	74.2	98.9	75.8	81	38.2	44.2
Patient 4_Met	99.9	60.9	57.5	23.2	75	33.1	43.8
Patient 4_WBC	99.7	14.1	82.4	22.1	17	38.3	38.4
Patient 5_CTC	99.8	73.4	98.4	70.8	68	38.1	43.5
Patient 5_WBC	98.8	15.7	87.2	21.7	17	38.1	40.0

**Table 3:** Common actionable genes found in CTCs and FFPE metastatic tissues.

		CTCs			Metastases		
Patient	Gene	Mutation Type	Protein Sequence Change	VAF (%)	Mutation Type	Protein Sequence Change	VAF (%)
Patient 1	SLIT2	Frameshift	p.S1686fs*2	0.12	Missense	p.I94T; p.I8T	0.07
	FAT1	Missense	p.H2768R; p.H2770R	0.44	Missense	p.H2768R; p.H2770R	0.75
					Missense	p.D4070N; p.D4072N	0.20
					Missense	p.P1349S	0.03
	PTCH1	Frameshift	p.S1203fs*17; p.S1052fs*17; p.S1137fs*17; p.S1202fs*17; p.S1151fs*17	0.05	Synonymous	p.E200E; p.E134E; p.E199E; p.E49E	0.29
	CHD2	Frameshift	p.A253fs*8	0.08	Synonymous	p.E23E	0.04
					Missense	p.G685S	0.06
Patient 3	SPTA1	Stop gain	p.Q340*	0.09	Missense	p.H2293N	0.30
					Missense	p.M121I	0.50
	CDC73	Missense	p.T334I; p.T253I	0.03	Missense	p.R171G	0.06
	LRP1B	Frameshift	p.H4368fs*6	0.14	Synonymous	p.C4299C	0.55
					Synonymous	p.F2419F	0.18
					Missense	p.F2419I	0.18
					Missense	p.D1807E	0.11
					Missense	p.T1147S	0.42
					Missense	p.V652L	0.12
	STAT4	Synonymous	p.N59N	0.07	Missense	p.A117T	0.62
	NSD1	Missense	p.D1466N; p.D1197N	0.09	Synonymous	p.L207L; p.L476L	0.19
		Missense	p.A1645T; p.A1376T	0.05	Stop gain	p.S478*; p.S209*	0.19
					Missense	p.R890Q; p.R1159Q	0.19
					Missense	p.R1608C; p.R1877C	0.09
					Missense	p.V1699D; p.V1968D	0.09
	ROS1	Missense	p.664R	0.07	Missense	p.P1133Q; p.P1138Q	0.14
					Missense	p.E1062K; p.E1057K	0.05
	GRM3	Missense	p.H94Y	0.04	Missense	p.I578N	0.33
					Missense	p.A609V	0.21
	MET	Synonymous	p.Q165Q	0.06	Missense	p.Y369H	0.14
					Missense	p.M33I; p.M463I	0.43
					Missense	p.C770Y	0.21
	FANCF	Missense	p.P76S	0.05	Synonymous	p.L321L	0.43
	CHD2	Missense	p.897T	0.08	Missense	p.P208T; p.P195T; p.P159T	0.12
	GNAS	Frameshift	p.V118fs*23; p.V102fs*23; p.V746fs*23; p.V103fs*23; p.V117fs*23; p.V760fs*23; p.V58fs*23	0.10	Missense	p.R150H; p.R106H; p.R166H; p.R808H; p.R151H; p.R794H; p.R165H	0.12
	KDM6A	Stop gain	p.Q753*; p.Q457*; p.Q760*; p.Q781*; p.Q708*; p.Q805*; p.Q736*; p.Q684*; p.Q702*; p.Q674*	0.02	Missense	p.R649L; p.R628L; p.R604L; p.R570L; p.R542L; p.R576L; p.R673L; p.R552L; p.R325L; p.R621L	0.25
					Stop gain	p.E1062*; p.E993*; p.E941*; p.E931*; p.E965*; p.E1038*; p.E1017*; p.E714*; p.E959*; p.E1010*	0.15
	ATRX	Missense	p.K1294E; p.K1332E	0.03	Synonymous	p.Q1603Q; p.Q1565Q	0.50
					Stop gain	p.E1231*; p.E1193*	0.06
					Missense	p.N1176K; p.N1214K	0.34
					Synonymous	p.D705D; p.D667D	0.10
					Missense	p.L582M; p.L620M	0.11
	CSF3R	Frameshift	p.S469fs*5	0.10	Missense	p.G487A	0.75
	REL	Missense	p.P580S; p.P548S	0.03	Missense	p.G347R; p.G379R	0.12
	DROSHA	Frameshift	p.C96fs*33	0.03	Missense	p.L804V; p.L767V	0.07
					Missense	p.W486C; p.W523C	0.33
	MSH3	Missense	p.G485D	0.06	Synonymous	p.L601L	0.07
					Missense	p.Q750H	0.45
	NCOR1	Synonymous	p.G1553G; p.G1569G; p.G1458G	0.05	Missense	p.S2341G; p.S2335G; p.S2438G	0.33
		Missense	p.S787N; p.S771N; p.S678N	0.07	Missense	p.P1317R; p.P1426R; p.P1410R	0.42
					Stop gain	p.R305*; p.R414*	0.77
					Missense	p.S103P	0.06
					Missense	p.D100N	0.08
	PIK3C3	Frameshift	p.T790fs*4; p.T727fs*4	0.04	Missense	p.Q860E; p.Q797E	0.13
	NCOA3	Missense	p.P261S	0.11	Missense	p.R198P	0.27
					Missense	p.Q496K; p.Q506K	0.43
					Missense	p.A511E; p.A501E	0.28
					Synonymous	p.L761L; p.L751L	0.43
					Missense	p.S794Y; p.S804Y	0.27
					Missense	p.Q1123E; p.Q1118E	0.03
					Missense	p.P1405R; p.P1409R; p.P1400R; p.P1408R	0.62

**Table 4:** Clinicopathological information of breast cancer patients.

Patient	Sample ID	Histology	ER	PR	HER2	Metastatic Site	Genomic or Genetic Testing Results	Timeframe between FFPE Biopsies and CTCs Collection
1	19065	Invasive ductal carcinoma (IDC)	Y	N	N	Liver	**FoundationOne:** Genomic alterations with associated therapies having potential clinical benefit: (1) PIK3CA (C420R)- Everolimus, Temsirolimus (2) CGN-NTRK1 fusion-Crizotinib (3) CHD4 loss exons 3–40 (4) CHEK2 M1T	5 days
2	78440	IDC	Y	Y	N	Pleural effusion	Germline BRCA2 mutation carrier	39 months
3	79130	IDC	Y	Y	N	Breast	None	29 months
4	80192	IDC	Y	N	N	Brain	None	19 months
5	28412	IDC	N	N	Y	Brain metastasis, unavailable for research	None	-

Y-Yes, N-No.

## Supplementary Materials

Supplementary materials can be found at https://www.mdpi.com/1422-0067/21/14/4826/s1.

## Abbreviations

ACMG	American College of Medical Genetics
AMP	Association for Molecular Pathology
ASCO	American Society of Clinical Oncology
ATCC	American Type Culture Collection
BCS	Broad copy number score
CAP	College of American Pathologist
CCDS	Consensus Coding Sequence Databases
CGH	Comparative Genomic Hybridization
CHOP	Children’s Hospital of Philadelphia
CNAs	Copy number alterations
COSMIC	Catalogue of Somatic Mutations in Cancer
CTCs	Circulating Tumor Cells
cfDNA	Cell-free DNA
ctDNA	Circulating tumor DNA
DMEM	Dulbecco’s Modified Eagle Medium
ExAC	Exome Aggregation Consortium
FCS	Focal copy number score
FDA	Food and Drug Administration
FDR	False discovery rate
FFPE	Formalin-fixed paraffin-embedded
FISH	Fluorescence in-situ hybridization
GATK	Gene Analysis Tool kit
gnomAD	Genome Aggregation Database
GSC	Global copy number score
HGMD	Human Gene Mutation Database
IDC	Invasive ductal carcinoma
IGV	Integrative Genomic Viewer
IPA	Ingenuity Pathway Analysis
IRB	Institutional Review Board
IVA	Ingenuity variant analysis
LAC	Los Angeles County
MBC	Metastatic Breast Cancer
MDA	Multiple Displacement Amplification
NGS	Next-generation sequencing
NK	Natural killer
OS	Overall survival
PBMCs	Peripheral blood mononuclear cells
PBS	Phosphate buffer saline
PFS	Progression-free survival
PoN	Panel of normal
SCNAs	Somatic copy number alterations
SRA	Sequencing Read Archive
SNPs	Single nucleotide polymorphisms
SNVs	Single nucleotide variants
SSVs	Somatic structural variants
SVs	Structural variants
TCGA	The Cancer Genome Atlas
TNBC	Triple negative breast cancer
USC	University of Southern California
VAFs	Variant allele fractions
WBCs	White blood cells
WES	Whole Exome Sequencing
WGA	Whole Genome Amplification
WGS	Whole Genome Sequencing
