# Supplementary material for: A Pilot Study for the Feasibility of Exome-Sequencing in Circulating Tumor Cells Versus Single Metastatic Biopsies in Breast Cancer
Source: Int J Mol Sci. 2020 Jul 8;21(14):4826. doi: 10.3390/ijms21144826 (PMC7402350; doi:10.3390/ijms21144826)
Supplement: Supplementary file 1 [file ijms-21-04826-s001.zip › supplementary figures.pdf]

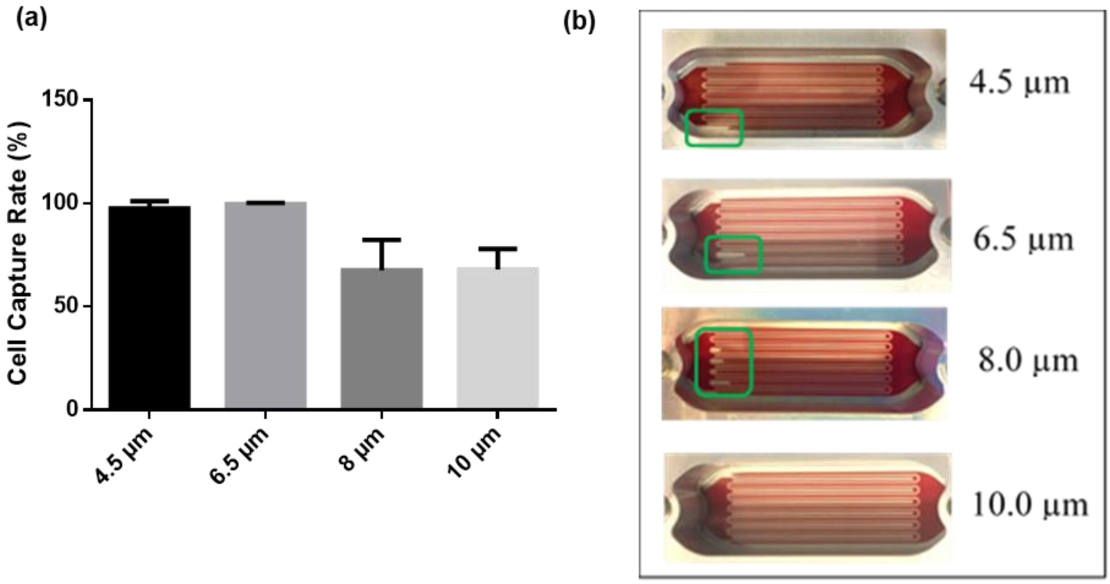

Supplementary Figure S1: Cell capture rate and priming efficiency of four different size cassettes (**a**) Bar graph showing the cell capture rate of four different size Parsortix cassettes. (**b**) Priming efficiency of four different size cassettes. The green highlighted region in the 4.5um, 6.5um, and 8um cassettes show insufficient priming in comparison to the 10um cassette.

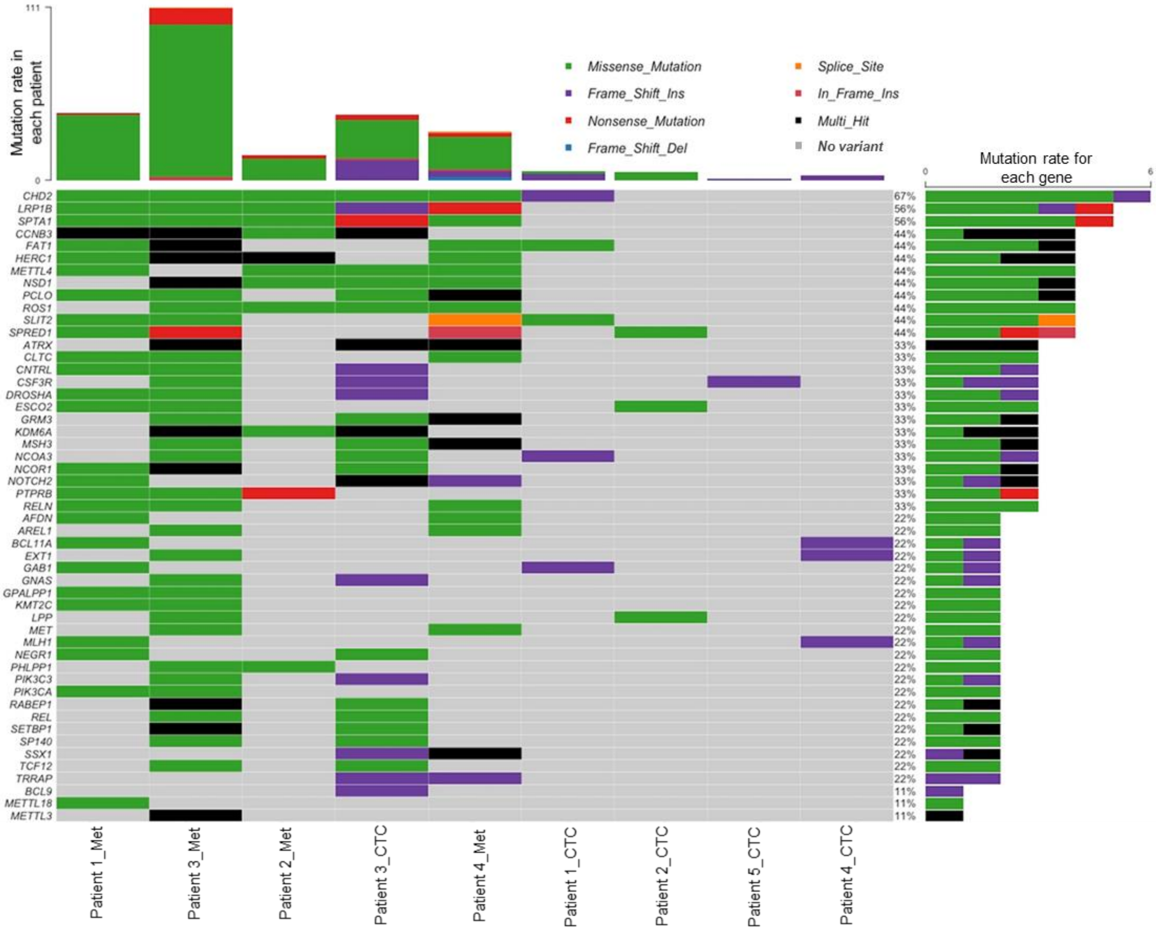

Supplementary Figure S2: Oncoplot showing the mutational pattern of 51 actionable genes that showed overlap in the FoundationOne, OncoKB, and MSK-IMPACT gene panel in CTCs and

metastases. The percentage to the right of the oncoplot shows the percent of samples with variants for the corresponding gene. Right, barplot shows the mutation rate in each of 51 potentially clinically actionable genes; Top, barplot shows the mutation rate for each patient for 51 actionable genes. By default, samples are ordered by the most frequently mutated genes.
